# Never‐Ending Learning for Explainable Brain Computing

**DOI:** 10.1002/advs.202307647

**Published:** 2024-04-11

**Authors:** Hongzhi Kuai, Jianhui Chen, Xiaohui Tao, Lingyun Cai, Kazuyuki Imamura, Hiroki Matsumoto, Peipeng Liang, Ning Zhong

**Affiliations:** ^1^ Faculty of Engineering Maebashi Institute of Technology Gunma 371–0816 Japan; ^2^ School of Psychology and Beijing Key Laboratory of Learning and Cognition Capital Normal University Beijing 100048 China; ^3^ Faculty of Information Technology Beijing University of Technology Beijing 100124 China; ^4^ Beijing International Collaboration Base on Brain Informatics and Wisdom Services Beijing 100124 China; ^5^ School of Mathematics, Physics and Computing University of Southern Queensland Toowoomba 4350 Australia

**Keywords:** evidence combination and fusion computing, explainable brain computing, functional neuroimaging, high‐order brain cognition, human‐in‐the‐loop, never‐ending learning, thinking and reasoning

## Abstract

Exploring the nature of human intelligence and behavior is a longstanding pursuit in cognitive neuroscience, driven by the accumulation of knowledge, information, and data across various studies. However, achieving a unified and transparent interpretation of findings presents formidable challenges. In response, an explainable brain computing framework is proposed that employs the never‐ending learning paradigm, integrating evidence combination and fusion computing within a Knowledge‐Information‐Data (KID) architecture. The framework supports continuous brain cognition investigation, utilizing joint knowledge‐driven forward inference and data‐driven reverse inference, bolstered by the pre‐trained language modeling techniques and the human‐in‐the‐loop mechanisms. In particular, it incorporates internal evidence learning through multi‐task functional neuroimaging analyses and external evidence learning via topic modeling of published neuroimaging studies, all of which involve human interactions at different stages. Based on two case studies, the intricate uncertainty surrounding brain localization in human reasoning is revealed. The present study also highlights the potential of systematization to advance explainable brain computing, offering a finer‐grained understanding of brain activity patterns related to human intelligence.

## Graphical abstract

To advance the understanding of human intelligence, this study presents an explainable brain computing framework in a scalable never‐ending learning paradigm with human‐in‐the‐loop. Integrated units manage interaction, learning, and resources for systematic research across diverse knowledge, information, and data sources. Case studies validate the causal and uncertain effects of brain localizations on high‐order cognition.

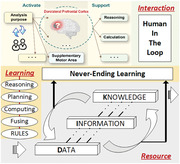

## 1 Introduction

Artificial intelligence (AI) is increasingly integrated into the study of decoding brain cognition, aiming to clarify high‐order cognitive functions with complex brain mechanisms, especially to meet the high requirements on explainability. While recent research has concentrated on large‐scale and multi‐view neuroimaging analyses to enhance confidence and explainability, effectively synthesizing findings from various separate investigations over time remains a challenge. This hinders the comprehensive expression and global interpretation of complex brain cognition.^[^ ^1^, ^2^, ^3^ ^]^ To address this challenge, refining fusion computing on multi‐source knowledge, information, and data is necessary to improve joint learning from diverse evidence. This approach can contribute to understanding the nature of human intelligence, brain function, and behavior while advancing the development of brain‐inspired intelligence technology for realizing human‐level AI society. To achieve this goal, brain computing is performed to uncover human intelligence, with the core task of decoding the many‐to‐many mapping relationships between brain patterns and cognitive functions. For instance, a specific brain activity pattern may relate to a variety of cognitive functions and a specific cognitive function may be identified as a number of brain activity patterns.^[^ ^4^, ^5^, ^6^ ^]^ Especially for high‐order cognitive functions, such as the key components of inductive reasoning, rule identification and rule extrapolation are found to be highly correlated to the dorsolateral prefrontal cortex (DLPFC).^[^ ^7^, ^8^, ^9^ ^]^ However, it is also reported that the DLPFC is significantly relevant to other cognitive functions with delicate uncertainty and high unexplainability, such as calculation, decision‐making, problem‐solving, and working memory.^[^ ^10^, ^11^, ^12^ ^]^ Therefore, new brain computing approaches are needed from systematic, robust, and explainable viewpoints to interpret the relative specificity of brain activity patterns to various cognitive functions.^[^ ^13^, ^14^, ^15^ ^]^

Presently, the prevailing approaches for decoding brain cognition involve integrating multiple results from different investigative viewpoints. This process encompasses two analytical strategies: correlation and comparison across multiple cognitive domains.^[^ ^16^, ^17^, ^18^, ^19^ ^]^ These approaches play a crucial role in decoding the specificity and generality of brain patterns. Subsequently, they provide a uniform expression and global interpretation that can model numerous external stimuli experiments with both similar and different task characteristics. For instance, by repeating the same and similar experimental paradigm, an intra‐domain brain computing strategy can increase the confidence of results, while an inter‐domain brain computing strategy can elucidate the diversity of multiple cognitive components and brain patterns through performing different types of experimental paradigms. A key point in these strategies is the selection and prioritization of cognitive experiments with sophisticated design properties to enhance the explainability and interpretability of brain computing. Furthermore, these properties can be integrated into algorithms and machines to enable them to perform systematic experimental planning like human beings, wherein experimental evidence from different views can be systematically learned and used to support future brain computing studies.

To achieve such a brain computing method that can address the large‐scale and multi‐source fusion issues from various separate investigations systematically, existing analysis methods, such as meta‐analysis approaches,^[^ ^20^, ^21^ ^]^ have been widely used but with several limitations. First, such analyses mainly focus on a single data scale under a similar topic, such as peak coordinates of an investigative topic, which cannot provide sufficient explanations for multi‐scale computed results. For instance, peak coordinates of activated brain regions published in scientific articles corresponding to the same cognitive domain are extracted as a single data source, which can enhance statistical strength but cannot provide a global explainability from the view of multiple cognitive domains. Second, all fused data are analyzed equivalently at once, which is not continuous and neglects the delicate association of various evidence with implicate functions and experimental factors, insufficiently developing the systematic fusion of different types of evidence for decoding cognition. Third, it is challenging to integrate increasing resources flexibly, including multisource knowledge, information, and data, through previous experiences like human beings. For example, it is difficult to robustly integrate findings from new resources and accumulated diverse experience with previous knowledge, information, and data, and learn over time. All of these limitations prompt discussions on the explainability of brain computing processes and results. Those align with the current and future trends of advanced brain computing in cognitive neuroscience, that is the viewpoints of researchers are shifting toward a relative definition rather than an absolute one. This adjustment acknowledges the complexity of high‐order cognition within the brain, leading researchers to approach the explainability subject with a more nuanced viewpoint instead of seeking a definitive conclusion.

The pursuit of explainability in brain computing holds the potential to uncover the biological underpinnings of the human mind by enhancing the algorithmic transparency and the comprehension of high‐order cognition. Currently, explainable AI finds widespread applications in neuroscience and medical domains, spanning functional brain development, behavioral decoding, imaging biomarker analyses, and medicine.^[^ ^22^, ^23^, ^24^, ^25^, ^26^ ^]^ Especially, explainability can be strengthened during various stages corresponding to multiple objects of data, computing, and results in an AI method, which can be grouped into pre‐modeling explainability, interpretable model, and post‐modeling explainability.^[^ ^27^ ^]^ Previous evidence has shown that the pre‐modeling explainability can be strengthened by characterizing the input data such as dataset description, standardization, and summarization; the modeling explainability can be improved by designing an explainable model architecture and algorithm such as rule/decision sets and case‐based reasoning methods; and the post‐modeling explainability can be enhanced by extracting explanations from outputs such as explanation targets at different levels of uncertainty and macro‐explanations.^[^ ^28^, ^29^ ^]^ Inspired by recent advances in brain computing and explainability studies, we hypothesized that all of these strategies need to be taken into account jointly to support us to give a comprehensive observation of high‐order cognition through achieving explainable large‐scale brain computing analyses, together with expert preferences.

To support this notion, we contend that explainable brain computing encompasses not only the upscaling of data through a multi‐view integration of cognitive domains but also the integration of diverse data sources and experimental factors across various dimensions of analyses and tasks. This study introduces a novel explainable brain computing framework for the joint learning of multisource brain knowledge (K), information (I), and data (D) within a never‐ending learning paradigm, using a three‐layered KID architecture. This includes fundamental learning in separate knowledge, information, and data viewpoints, together with systematic operations in a higher meta‐learning viewpoint. On the one hand, we highlight the systematic organization and management of multiple resources from the data science view. On the other hand, we focus on the explainable operations of knowledge‐information‐data from the computing perspective, especially highlighting how to use these resources systematically to produce more explainable computing results. Accordingly, the framework realizes never‐ending learning by conducting evidence combination and fusion computing within a KID loop for systematic and explainable brain cognition decoding inspired by human thinking. Key features have been encoded into this framework as follows. First, it engages in internal evidence learning, conducting task‐state fMRI analyses based on both forward and reverse inference for specific hypotheses at the regional scale. Second, it incorporates external evidence learning by leveraging pre‐trained language modeling techniques to enrich the evidential scale, supporting the extension of decoding ideas from related neuroimaging scientific articles. Thirdly, it achieves evidence combination and fusion computing through systematic experimental planning and continuous integration of internal evidence from functional neuroimaging data and external evidence from neuroimaging articles. Consequently, the framework enhances layered and detailed explainability, facilitating the understanding of brain patterns from different cognitive domains through knowledge‐driven forward inference and the comprehensive analysis of cognitive functions from brain patterns through data‐driven reverse inference. These features collaboratively operate within a human‐machine interaction process, constituting a human‐in‐the‐loop mechanism for the never‐ending learning processes. This approach systematically integrates large‐scale task‐state fMRI and text data across diverse cognitive domains into a continuous investigation process when confronted with different cognitive hypotheses.

## 2 Results

### 2.1 Overview

**Figure 1:**
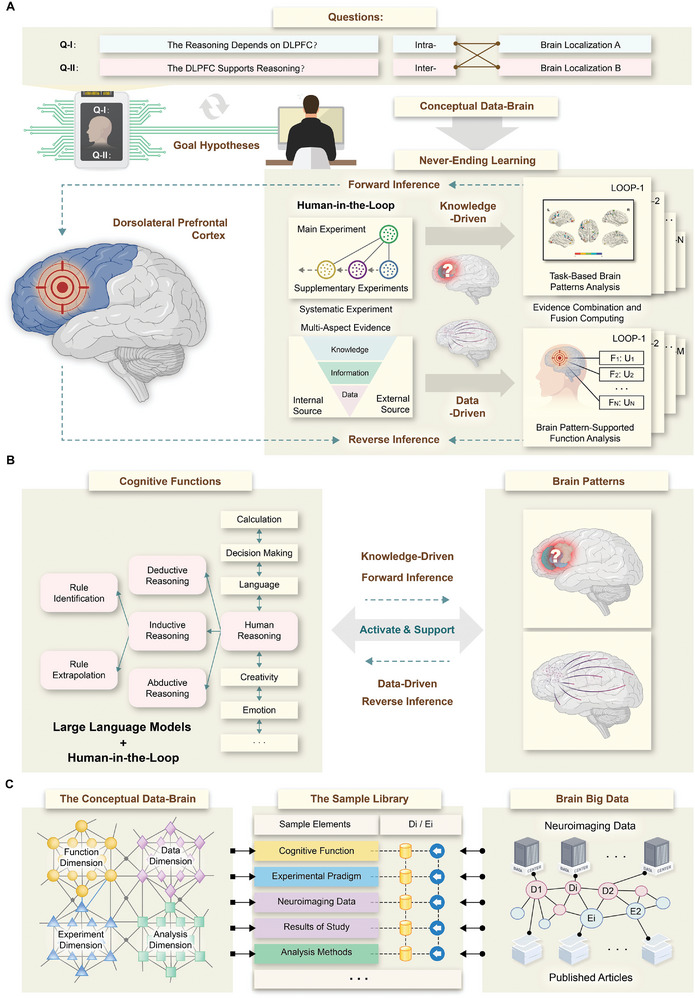
Schematic description of the never‐ending learning and its applications with the multidimensional brain data representation. A) The complex brain science problem is investigated by systematically testing various hypotheses. B) The concepts of cognitive functions and their relations from the conceptual Data‐Brain are rebuilt to generate a reasoning‐centric operation subgraph. C) The interconnected knowledge, information, and data are organized in the knowledge (K) – information (I) – data (D) architecture, producing a sample library to support explainable brain big data computing.

**Figure** **1** illustrates the explainable brain computing framework of never‐ending learning, which is found on a hierarchical architecture that relies on prior knowledge and rule reasoning for conducting a systematic exploration of brain functions and high‐order cognition (Figures S1 and S2; Table S1, Supporting Information). Within the architecture, the explainable learning process can be directed by systematic experiment planning, similar to how humans do it, in conjunction with both forward and reverse inference mechanisms. Additionally, it utilizes the human‐in‐the‐loop mechanism, ensuring that individuals can support the learning process in a simple yet impactful way.

To investigate the specific associations between the brain and cognition, the evidence combination and fusion computing approach is utilized to learn multi‐aspect and multisource functional neuroimaging resources, enabling the interpretation of both task‐activated brain patterns and brain pattern‐supported functions. As shown in Figure 1A, the core components and paradigms in the explainable brain computing framework are given. Herein, a complex brain science problem can be investigated by testing various hypotheses, for example, how a specific neuronal structure, such as the DLPFC, participates in a specific cognitive process, such as human reasoning, and exhibits stronger specificity among multiple cognitive processes. To achieve this goal, systematic brain computing highlights explainable experiments and multiaspect evidence to decode high‐order cognitive functions from both perspectives of forward and reverse inferences, ensuring intra‐ and inter‐analyses within evidence combination and fusion computing toward never‐ending learning. During the knowledge‐driven forward inference process, the task‐evoked brain activity patterns are analyzed to answer the Q‐I related questions. While the brain pattern‐supported function specificity is analyzed to answer the Q‐II related questions during the data‐driven reverse inference process. A detailed explanation of the framework and its workflows for exploring cognitive functions and brain activity patterns can be found in the following sections.

Figure 1B presents a generated case demonstrating the implementation of human reasoning‐centric systematic brain computing, as evidenced by sampling a personal subgraph obtained from the conceptual Data‐Brain that is a fragment of a global graph from the knowledge layer. In the current case study, the connected highlighted cognitive functions in pink boxes represent intra‐domain relations, while the inter‐domain relations are represented between the cognitive functions in pink boxes and those highlighted in orange boxes. Meanwhile, each cognitive concept connects one or multiple brain resources obtained by various cognitive tasks with various experimental and analytical elements. Based on the personal subgraph as a clue, the reasoning‐centric resources were integrated and processed to support evidence combination and fusion computing, thereby being learned to generate the explainable results during the knowledge‐driven forward inference and data‐driven reverse inference processes. Finally, the causal effects of brain patterns and cognitive functions are interpreted via the learned τ and γ distributions.

In Figure 1C, multi‐view evidence resources, encompassing knowledge, information, and neuroimaging data, have been integrated into a sample library to support explainable brain computing operations within the three‐layered KID architecture (Tables S1–S3, Supporting Information). The knowledge layer represents the complete process of systematic brain investigation from four dimensions: brain functions, experimental tasks, data management, and analytical methods. The information layer records provenance information and procedural details, which are derived from neuroimaging metadata and published research articles. The data layer integrates multimodal and multiscale brain big data from both perspectives of internal and external evidence (including the neuroimaging data as the internal evidence, Di, and the published articles as the external evidence, Ei, with details in Table S1, Supporting Information), both of which align with the conceptual Data‐Brain, such as original neuroimaging data, procedural data, and study results, along with their respective metadata.

**Figure 2:**
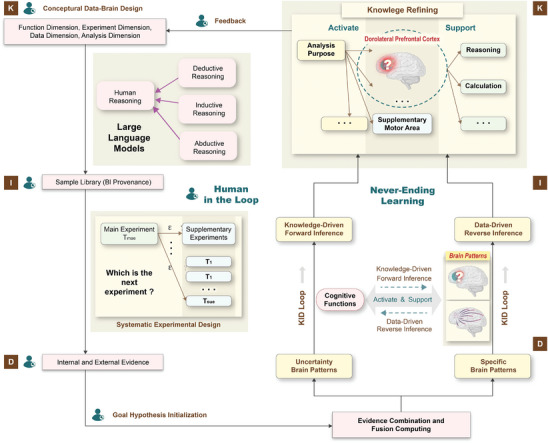
Illustration of the Knowledge (K)‐Information (I)‐Data (D) loop and its key components during the never‐ending learning, together with human‐in‐the‐loop.

As shown in **Figure** **2**, the workflows of the framework involve several core steps. In particular, the implementation of never‐ending learning relies on the KID loop within the three‐layer KID architecture, which continuously iterates and fuses aligned computing results from multiple sources of knowledge, information, and data. As the foundations, the conceptual Data‐Brain in the knowledge layer is learned by integrating pre‐trained language modeling techniques, which is designed to transform large‐scale text data to domain‐specific knowledge graphs as a global graph, providing prompts during never‐ending learning and human‐in‐the‐loop processes. The types and weights of evidence are learned in the information layer to execute the internal and external evidence combination and fusion computing operations constrained by such a personal subgraph. In the data layer, multisource data are represented by provenances of the information layer, and then mapped and aligned to the global graph of the knowledge layer. During the dynamic learning stages, investigators formulate questions related to cognitive functions and brain patterns to trigger the never‐ending learning process, in combination with the human‐in‐the‐loop mechanism. For example, the effect of reasoning on DLPFC can be investigated using the framework.

Through the goal hypothesis initialization, computational parameters such as the number of loops and algorithmic details are determined from the experimental and analytical factors of interest. The multiaspect evidence is then sampled and integrated with an evidence combination and fusion computing approach, followed by systematic experimental planning. Similar to the thinking processes of human beings, that is how to design the next experiment to support systematic and continuous hypothesis testing? For a machine, it performs programs to address the issues of which is the next experiment, which is determined by the predefined rules‐based data selection and sampling methods, as shown in Section 4 for details on evidence combination and fusion computing. In this step, related experiments are determined by running various inference rules that correspond to different experimental types, including the main and supplementary experiments (Figure S2, Supporting Information). Moreover, the functional neuroimaging data obtained from these experiments and related scientific articles are systematically analyzed using intra‐ and inter‐computing strategies to enhance understanding of cognitive functions in brain localization. In addition, preferences and individuals are important characteristics to understand the brain and cognition. Facing such complex cognitive neuroscience issues, we contend that human factors are essential to support and supervise the learning processes, compared with the fully automation mode. In Figure 2, it can be found that the human‐in‐the‐loop mechanism is embedded into the framework with the human signs.

Furthermore, a loop includes two core schemes, namely, knowledge‐driven forward inference and data‐driven reverse inference. On the one hand, the former performs the mapping process from the hypothesized brain functions (reorganized knowledge and information) to multiview evidence combination and fusion computing. During this process, through fusing the brain patterns of various cognitive functions, the τ maps are computed to interpret the generality of various brain patterns for a hypothesized cognitive function. On the other hand, the latter performs the mapping process from a specific brain pattern (computed by reorganized data and information) to various brain functions (knowledge refining). During this process, the support coefficients γ of the brain patterns on various cognitive functions are computed to evaluate the extent to answer which a specific brain pattern supports different cognitive processes significantly. Similarly, the human‐in‐the‐loop mechanism is utilized in each step of the KID loop as needed to provide interactive input or qualitative evaluations.

In this study, to gain a comprehensive understanding of human reasoning, reasoning‐centric intra‐ and inter‐cognitive components were analyzed from the brain localization perspective. For this, multitype resources were integrated and learned, including four‐dimentional concepts of the knowledge layer in the conceptual Data‐Brain, twelve categories of neuroimaging entities, and fifty‐five categories of neuroimaging entity interactions in the information layer, as well as six task‐state fMRI datasets and over ten thousand scientific articles in the data layer (Tables S1–S5, Supporting Information). These neuroimaging data, serving as internal evidence, were prioritized in loops during never‐ending learning (Section 4 for details on internal evidence learning). Additionally, thirty‐two reasoning‐related neuroimaging studies were sampled as external evidence from over seven hundred neuroimaging‐related full‐text articles using the BI (Brain Informatics^[^ ^30^ ^]^) provenance‐based neuroimaging topic modeling method (Section 4 for details on external evidence learning). The reported results in external evidence further contributed to never‐ending learning, thereby extending the learning scale. Moreover, the identified topics and their interactions could be utilized to refine and explain the specificity and generality of high‐order cognition. In the following parts, the human reasoning‐related results are given during the never‐ending learning process.

### 2.2 Never‐Ending Learning of Integrating the Forward and Reverse Inference for Systematic Understanding of Human Reasoning

**Figure 3:**
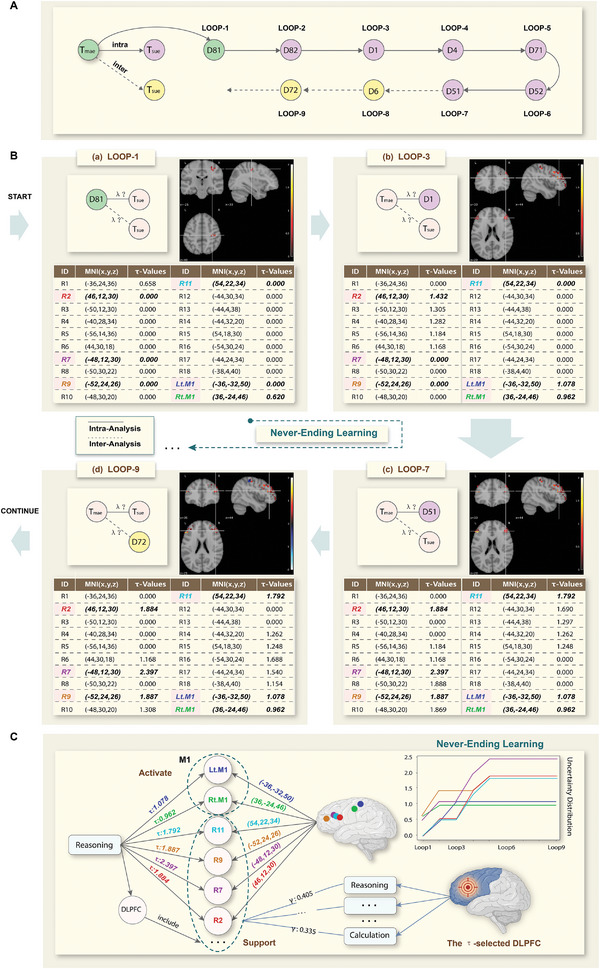
Understanding the brain mechanism of reasoning based on the never‐ending learning from the brain region perspective. A) The generated experiment sequence is shown based on the internal evidence, toward never‐ending learning of reasoning within hypothesized brain regions. B) The changes in the forward inference actions occur in each loop and have an impact on the interpretations of the outcomes given by different loops, where the selected results are shown in this figure. C) The left part gives the results from the knowledge‐driven forward inference, showing that some clusters in the DLPFC are significantly activated by the reasoning‐oriented tasks. The right part gives the results from the data‐driven reverse inference, showing that the DLPFC provides relatively stronger support for human reasoning in contrast to calculation. The integrated results from the forward and reverse inference prompted us to causally correlate reasoning with the DLPFC.

To validate the explainable brain computing framework, we conducted a scenario to test the hypothesis regarding the role of DLPFC in human reasoning (**Figure** **3**). The conceptual Data‐Brain outlines the reasoning‐centric cognitive subcomponents and their relationships with other domain dimensions (Figure S1, Supporting Information). Investigators determined the experimental and analytical factors, including reasoning in the function dimension, factorial and block design in the experiment dimension, task‐state fMRI data in the data dimension, and statistical parametric mapping and machine learning in the analysis dimension, based on the human‐in‐the‐loop mechanism that reflects their specific interests. The never‐ending learning process was guided by systematically planning experiments and continuously sampling various types of evidence from the sample library, which generates multiple loops, such as D81 in LOOP‐1, D1 in LOOP‐3, D51 in LOOP‐7, and D72 in LOOP‐9 (Figure 3A for details of loops).

During the knowledge‐driven forward inference process, the task‐state fMRI data as internal evidence were analyzed and extracted from the sample library to measure the effect size of reasoning on regions of interest, including the DLPFC and the primary motor cortex (M1) as the control region, through computing parametric brain maps.^[^ ^31^ ^]^ Next, the τ‐maps are generated by the evidence combination and fusion computing approach (Section 4 for details on evidence combination and fusion computing), which continuously updates the reasoning‐related brain patterns during this knowledge‐driven process. For each loop, the voxel clusters can be obtained in two ways: one is obtained by performing the general linear model methods to compute statistical parametric maps; another is obtained by the reverse computation extracted by studies from selected coordinates to brain maps.

We extracted the clusters with larger τ values compared with the maximum τ‐value in M1, including Lt.M1 and Rt.M1 (Figure 3B). In the present case, the D81 sample is the main experiment, as the starting point of never‐ending learning. The rest of the samples are the supplementary experiments, where D1 and D51 are identified as Type‐I evidence and D72 is identified as Type‐II evidence. In each loop, the sample was computed by statistical parametric mapping and then converted to τ‐Values to fuse the results from the previous loops. Accordingly, the Type‐I evidence was fused to select brain regions showing specificity for reasoning from LOOP‐1 to LOOP‐7, such as the prefrontal cortex.^[^ ^32^ ^]^ Furthermore, the Type‐II evidence was fused from LOOP‐8 to LOOP‐9 to reduce uncertainty created by other functional domains, such as calculation. It can be found that the clusters changed from the peak coordinates (such as R1 in LOOP‐1) to other peak coordinates (from R2 to R6 in LOOP‐3). As a result of the staged never‐ending learning process, reasoning‐specific brain patterns were explained in LOOP‐9, including the first four clusters (two in the left hemisphere and two in the right hemisphere) with the maximum peak values in the DLPFC (the left side of Figure 3C). Herein, the first four clusters with the maximum τ‐Value were observed in R2, R7, R9, and R11. The left primary motor area, i.e., Lt.M1, and the right primary motor area, that is, Rt. M1 was taken as a control brain region.

During the data‐driven reverse inference process, the support coefficient γ of brain patterns of interest was computed to measure the effect size of those on various cognitive processes, such as reasoning and calculation, using information‐based mapping.^[^ ^33^ ^]^ Initially, without considering the lateralization of the left and right DLPFC, the support coefficient γ = 0.405 of the brain pattern selected by the τ‐map (τ > the maximum peak value of τ in M1) for reasoning was found to be higher than that for calculation (γ = 0.335), as shown on the right side of Figure 3C. Next, the lateralization of the left and right DLPFC was considered to evaluate the support coefficients of the τ‐selected brain patterns. The left DLPFC brain pattern could attain a support coefficient of 0.346 for reasoning and 0.311 for calculation, whereas the right DLPFC brain pattern could attain a support coefficient of 0.397 for reasoning and 0.325 for calculation. Additionally, the support coefficients of the first four clusters selected by the τ‐map for reasoning (R7 and R9 in the left DLPFC and R2 and R11 in the right DLPFC) were evaluated. The integrated R7 and R9 in the left DLPFC could attain a support coefficient of 0.407 for reasoning and 0.327 for calculation, while the integrated R2 and R11 in the right DLPFC could attain 0.400 for reasoning and 0.325 for calculation.

### 2.3 Never‐Ending Learning of Integrating the Internal and External Evidence on the Core Brain Regions of Human Reasoning

**Figure 4:**
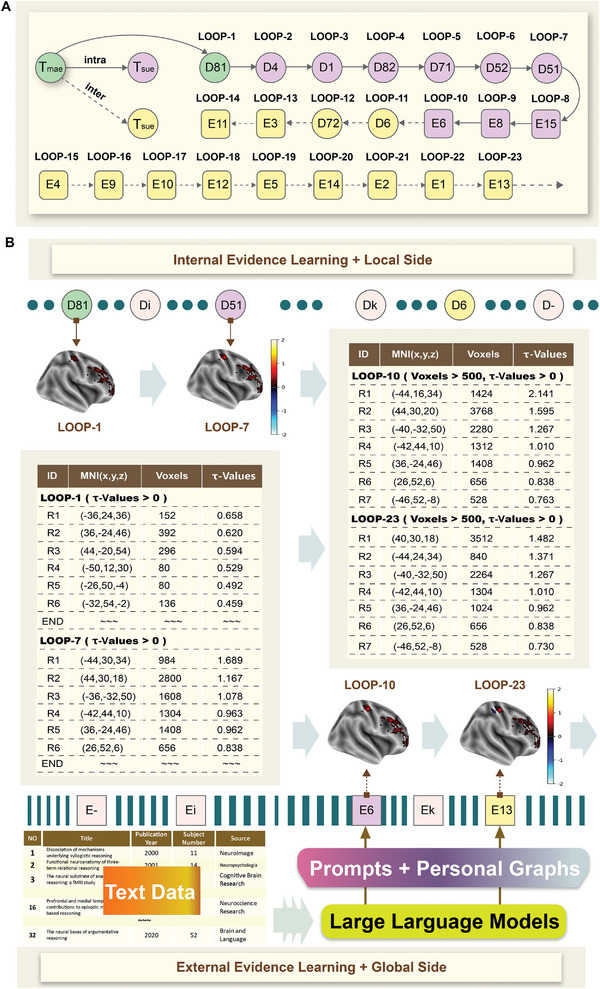
The never‐ending learning of integrating internal and external evidence for systematic understanding of human reasoning. A) The whole planned experiment sequence is shown based on the combination of internal and external evidence toward the never‐ending learning of human reasoning. The planned internal and external evidence is extracted from the sample library (Tables S1 and S4, Supporting Information). B) Based on internal evidence learning, some finer‐grained changes to the clusters and their uncertainty distributions can be found during the fusion computing of external evidence, where the selected results are shown in this figure.

The extensible capabilities of never‐ending learning were put to further test by integrating multisource and multilevel evidence from internal and external views. On the one hand, the internal evidence was obtained from the sample library which contained six task‐state fMRI datasets collected from over 100 subjects. On the other hand, the external evidence was derived from published neuroimaging articles. A total of 677 open‐access full‐text neuroimaging‐related articles, published between July 2014 and July 2019, were crawled from the PLOS series of journals using relevant keywords, such as “reasoning”. In addition, 44 neuroimaging articles that focused on reasoning were downloaded from PubMed, based on published time and keywords. The BI provenance‐based neuroimaging topic modeling method was employed to identify the experimental and analytical factors in each article (detailed factors in Tables S2 and S3, Supporting Information). As presented in **Figure** **4**, the text data of each neuroimaging article could be extracted as a BI provenance, consisting of a group of factors (neuroimaging topics). The extracted BI provenances, including factors and reported results, were stored in the sample library for evidence combination and fusion computing. During the testing stage, reasoning‐related evidence was extracted based on systematic experimental planning with similarity assessment. Thirty‐two articles were matched as supplementary experiments (Table S4, Supporting Information), including their three types of representative factors of experimental paradigms, experimental protocols, and explicit stimuli (Table S5, Supporting Information).

The entire learning process comprised of the following steps: (1) utilizing external evidence learning, through the BI provenance‐based neuroimaging topic modeling method, to identify multiple factors in experiments, analyses, and reported results from published neuroimaging articles, and storing them in the sample library; (2) extracting evidence related to the goal hypothesis, such as reported coordinates, from the sample library based on systematic experimental planning; (3) tracking and reconstructing original data extracted from external evidence to the greatest possible degree; and (4) performing alignment and fusion computing of internal and external evidence to verify and enhance the understanding of human reasoning. The external evidence is continuously added after internal evidence learning, expanding the observed views and boosting the confidence of the learned results (the detailed loops illustrated in Figure 4A).

Figure 4B illustrates some of the learned results in relation to human reasoning. Herein, internal evidence learning affects from LOOP‐1 to LOOP‐7, LOOP‐11, and LOOP‐12, while external evidence learning affects from LOOP‐8 to LOOP‐10, and LOOP‐13 to LOOP‐23. To observe all changes that occurred during the never‐ending learning process, the learned τ‐Values in the peak coordinates selected from the last loop, LOOP‐23, were examined throughout all the learned loops (Table S6, Supporting Information). The computed τ‐maps were found to change from LOOP‐1 to LOOP‐23 as the use of internal and external evidence incrementally. In the last loop, the selected top clusters in the DLPFC were reported based on the intensity of the peak τ‐Values. The τ‐Values of R1 and R2 were found to be larger than that of the max τ‐Value in M1 (i.e., the control area), indicating that they were more closely related to reasoning. These results were obtained through the extensive use of external evidence from the eleventh loop onwards. To increase the confidence in the results obtained by internal evidence learning, only results with τ‐Values > 0 and Voxels > 500 were considered. τ‐Values > 0 means that the selected results are related to the hypotheses, while Voxels > 500 is dependent on individual preference.

Furthermore, due to the effects of inter‐type evidence, the τ‐Values of some regions such as R7 decreased from LOOP‐10 to LOOP‐23, indicating that these regions had weak cognitive specificity for human reasoning. On the other hand, some new regions were generated through continuous evidence learning, which had a positive impact on human reasoning. Notably, the number of clusters that matched the selection conditions of peaks increased from 0 in LOOP‐1 to 6 in LOOP‐7 and 7 in LOOP‐10 and LOOP‐23. Herein, D81 in LOOP‐1 and D51 in LOOP‐7 belong to the internal evidence type, while E6 in LOOP‐10 and E13 in LOOP‐23 are gathered by reported results from selected articles using external evidence learning, belonging to the external evidence types. All of them were extracted and computed systematically through prompts and personal graphs.

## 3 Discussion

In this study, we developed a novel brain computing framework that utilizes a never‐ending learning paradigm in combination with a human‐in‐the‐loop mechanism. Never‐ending learning is a typical paradigm in the AI field, which has a similar concept to a series of specific methods like incremental learning (also referred to as lifelong learning, continuous learning, and continual learning).^[^ ^34^, ^35^, ^36^, ^37^ ^]^ In the current study, we enlarge its functions on enhanced systematization, robustness, and explainability. Accordingly, the framework allows us to explore the relative specificity relationships between cognitive functions and brain patterns, thus enhancing our ability to decipher the neural basis of complex human cognitive functions, together with expert preference.

We demonstrated the effectiveness of the explainable brain computing framework through several case studies, aimed at systematically understanding how human reasoning occurs in the brain. Depending on the proposed hypotheses, a series of experimental plans, together with the computational details, were generated during the increasing loops (Figure 3A and Figure 4A for detailed loops). These cases clearly demonstrated the specificity of human reasoning within the hypothesized brain region (i.e., DLPFC). On the one hand, the effectiveness of this underlying mechanisms has been verified during the forward inference process (Figure 3B and Figure 4B), and is highly beneficial for never‐ending learning. On the other hand, the explainable brain computing framework also provides a view to verify the causal effects through the joint forward and reverse inference processes. The brain computing results from the forward inference process exhibited a strong correlation between the DLPFC and reasoning, in contrast to the control region M1 (the left panel of Figure 3C). Conversely, the reverse inference process revealed a significantly greater support coefficient value on the DLPFC to reasoning than calculation (the right panel of Figure 3C). Taken together, multiview analyses based on the proposed framework thus provided strong evidence suggesting that the DLPFC plays a causal role in human reasoning. This result extends our previous findings, which reported a correlation between the DLPFC and reasoning by integrating as much worldwide experimental evidence as possible. Furthermore, the current outcomes could serve as a starting point for subsequent learning with the inclusion of larger resources during the never‐ending learning process in the future.

Unlike conventional brain analytical methods, such as meta‐analyses, the fundamental idea behind it comes from examining research outcomes across different studies with similar topics, measuring the same variables, and having comparable methodologies, as with randomized controlled trials. Accordingly, the meta‐analyses and their advanced methods are good at the homogenous resources such as processing large‐scale study results with the similar cognitive experiments and topics, even though the evidence with different weights. Our proposed brain computing framework highlights a never‐ending learning process, in which the computing operation is not at once, but heterogenous, incremental, and dynamic. First, the current framework can handle with heterogenous evidence with different properties and profiles to explain a cognitive hypothesis comprehensively, based on the integration of multiple homogenous computing operations. Second, the framework does not limit itself to modeling a single viewpoint of the functional domain, cognitive experiment, data source, or analysis method during the brain investigation process. Instead, it is guided by a unified strategy that combines knowledge‐driven forward and data‐driven reverse inference with fusion computing from multisource knowledge, information, and data to learn brain functional representations systematically. Thirdly, the never‐ending learning paradigm allows for bidirectional switching of learned patterns, and regulation of operated parameters at each step of the learning process. These unique features facilitate the generation and verification of new hypotheses by integrating worldwide knowledge, information, and data, as well as provide a powerful tool for investigating the brain cognition and advancing our understanding of its functional mechanisms.

**Figure 5:**
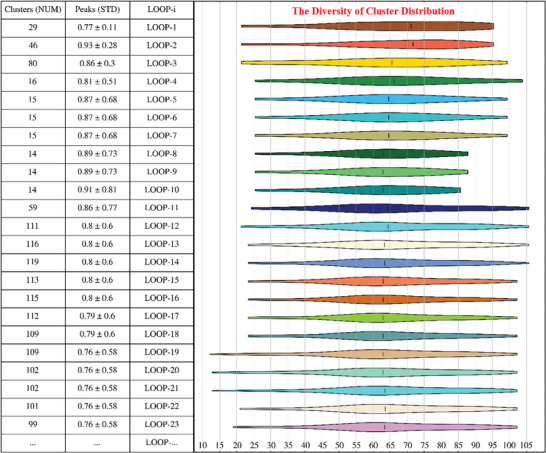
The evaluation of stability and plasticity for computed results during never‐ending learning within the explainable brain computing framework.

To achieve a balance between stability and plasticity, we adopt the “local‐ and internal‐evidence learning prior” principle. This principle prioritizes data with stronger relevance and richer metadata to predefined goal hypotheses for initial selection, integration, and computation. Our experimental observations suggest that the variations in clusters and coordinates across different loops effectively reflect the stability and plasticity inherent in our framework. To corroborate these findings, we conducted statistical analyses on the τ‐maps obtained across all loops, as illustrated in **Figure** **5**. In this figure, the left table's first column denotes the number of clusters, the second column displays the mean values and their respective standard deviations for selected peak values, and the third column specifies the loop index. The right section of the figure showcases the spatial diversity of cluster distributions, determined by the calculated distances between all peak coordinates and the origin coordinates. Our findings indicate that the number of clusters is approaching 100, peak values are trending toward 0.8 with a standard deviation of ≈ 0.6, and the diversity of cluster distributions falls between 60 and 65. In summary, our framework exhibits both stability, as evident from the left table, and computational plasticity, as observed in the right figure.

**Figure 6:**
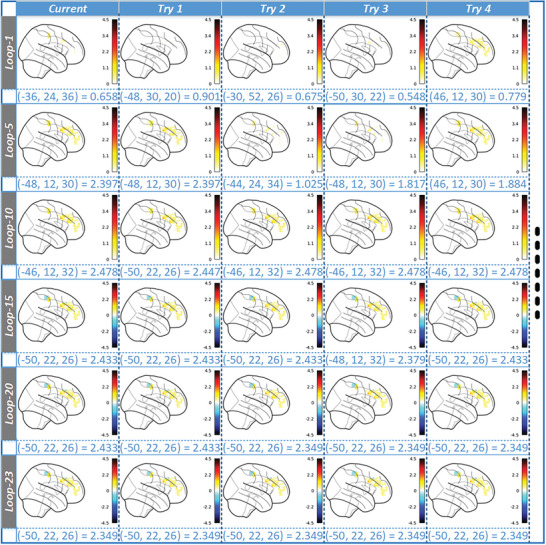
The observation and validation of bias from multiple simulations during never‐ending learning within the explainable brain computing framework.

Utilizing a human‐in‐the‐loop mechanism, our approach facilitates interactive learning with investigators across various uncertainty scales within a hypothetical space. This interactive process empowers us to define computing details and search scopes based on both internal and external evidence, while also constraining the never‐ending learning processes. Investigators can oversee and adjust each step during these loops, thereby ensuring that the generated output is unique for each individual preference based on their respective background knowledge and interests, even when facing the same research hypothesis. In particular, the rapid expansion of external evidence accelerates the iteration and updating of the conceptual Data‐Brain, ensuring the potential of the framework on continuous learning. Moreover, the design of automation strategies promotes the continuous integration of resources into the sample library, which further supports large‐scale brain studies. However, it is possible to bring about biases from learning processes. Accordingly, the key point lies in achieving a balance between bias and preference. That is to say, the finalized learned results remain stable and unchanged when maintaining the same initializing parameters and input resources, even though the sequence of loops can change the intermediate computing operations depending on individual preference. Facing this, one strategy for mitigating biases is to enhance the robustness of the framework. To verify the ability, we performed experiments that simulate the personal computing processes corresponding to different human preferences as shown in **Figure** **6**. In this figure, the results from the first column are associated with Figure 4, while those from the other columns are based on the random computing sequences. Throughout the five program runs, the largest peak appears in (−50, 22, 26) with the same τ‐Value of 2.349 from the final loop, while the reported results are different during the intermediate loops. By observing the results, it can be found that the finalized outcomes remain stable and unchanged when retaining the same initialization parameters and inputs, even with varying computing sequences of sampled resources. In conclusion, our framework demonstrates robustness in mitigating biases and maintaining stability, confirming its reliability and effectiveness.

In addition, we contend that the complexity of high‐order cognition determines the crucial issues of descriptive information, including better explainability from joint pre‐, mid‐, and post‐modeling studies, as opposed to relying solely on a single approach with limited goals and explainability. Specifically, it requires that a method/system should explain its abilities and understandings comprehensively—knowing what it has done, what it is doing now, and what will happen next; and disclose the salient information that it is acting on.^[^ ^38^ ^]^ To address this, the KID loop is designed to execute knowledge‐inspired and information‐constrained multisource data fusion computing, together with expert preference and rules. In the realm of data science and knowledge management, diverse definitions have been given to the concepts of knowledge, information, and data.^[^ ^39^ ^]^ In the present study, we emphasize the systematic organization and management of resources in the KID architecture from the data science perspective. Simultaneously, we further focus on the knowledge‐information‐data operations from the explainable computing perspective, particularly emphasizing how these resources contribute to producing more systematic and explainable computing results. Consequently, we integrate some text mining and semantic techniques to enhance the data explainability, a series of explicit rules to enhance the computing explainability, and uncertainty‐oriented evidence combination and fusion computing methods to enhance result explainability.

Generally, the explainable brain computing framework can work while the resources are operated and computed in a KID architecture to satisfy the following principles: a component to represent the commonsense knowledge that we would like to study, which inspires the users to propose hypotheses and guides the machines to execute computing; a component to systematically represent multi‐type resources that can be interconnected and used easily, which improves the data explainability and computability; a component to align data resources and processed results to make fusion computing available. Based on that, resource quality also plays an important role, especially in the large‐scale computing scenario. In an extreme case, if all input resources are homogenous, that is the computed resources have a similar property and inner mechanism, it will make learned results unchanged across different loops. Conversely, if all resources are entirely different, that is the computed resources have extremely high diversity, it can make results instability. To address this, we need to consider the impact of diversity by taking control of the operating rules. In particular, the framework prioritizes internal evidence with high confidence (from intra‐experiment to inter‐experiment evidence), followed by external evidence with relatively lower confidence. This sequential strategy allows the explicit design of rules and detailed methods, guiding the never‐ending learning process in analyzing multisource neuroimaging‐related resources for a nuanced understanding of high‐order cognition in different scenarios.

As a highly modular and scalable framework, explainable brain computing adeptly handles multi‐task functional neuroimaging studies during the never‐ending learning processes with acceptable robustness and stability (Supplementary Text in Supporting Information). However, in order to achieve a nuanced understanding of high‐order cognition, this proposed framework still has limitations in some specific scenarios. First, the current framework is limited to task‐based neuroimaging data and text. However, the mechanisms of cognitive functions need to be further explored to obtain more refined patterns on larger and broader scales. In the future, the framework should enhance its flexibility and alignment ability in integrating task‐free data,^[^ ^40^ ^]^ multiscale data,^[^ ^41^ ^]^ and so forth. Second, the current framework mainly focuses on understanding the mechanisms of high‐order cognition. However, understanding the interactions and associations among cognitive functions during conditions and diseases are other valuable topics, where the structural and functional alignment problem needs to be further investigated to reduce the gap between cognitive and clinical findings.^[^ ^42^, ^43^ ^]^ In the future, we aim to further confront challenges in translational research to make it available in clinical practices. In particular, other technologies are further integrated, such as brain stimulation, providing a novel perspective for decoding cognitive mechanisms.^[^ ^44^, ^45^, ^46^ ^]^ We encourage more scientists from different backgrounds to explore and recognize its value in various scenarios, contributing to a more profound understanding.

In a broader sense, our study may contribute to advancing the machine intelligence paradigm through decoding complex information‐processing mechanisms in the human brain. While single‐view learning is essential, it has limited utility in systematic understanding and multiaspect interpretations. Therefore, our framework, which integrates internal and external evidence, offers a promising direction for future investigations in the brain. It can further enhance evidence combination and fusion computing toward never‐ending learning, providing a more comprehensive and dynamic approach to understanding the complex brain mechanisms, and paving the way for further advancements in machine intelligence.

## 4 Experimental Section

In this section, we present the theoretical derivations and never‐ending learning mechanisms of the explainable brain computing framework. Detailed technical workflows and performance analyses can be found in Supporting Information.

### Never‐Ending Learning on Multi‐Source Brain Big Data Computing

The never‐ending learning paradigm utilizes existing knowledge, integrates big data from the brain, and guides the next steps of learning to ensure continuous updates and interpretations of brain computing results. In this study, the human‐centric KID loop was designed to drive never‐ending learning of human brain intelligence, performing the systematic investigation process through multisource integrated neuroimaging‐related knowledge, information, and data. The KID loop was a hypothesis‐triggered closed loop along with continued expansion of the conceptual Data‐Brain and brain resources. It includes knowledge‐driven forward and data‐driven reverse inferences involved in systematic experimental planning, intra‐ and inter‐analyses, evidence combination and fusion computing, and human‐in‐the‐loop interactive learning. During such a never‐ending learning process, the forward inference was performed to understand cognitive function‐related brain patterns at each loop, while the reverse inference was performed to validate the support coefficient of brain patterns to various cognitive functions, along with the incremental combination of new evidence.

In the first loop, a cognitive experiment was planned that aligned with the interests of investigators and drew upon relevant evidence from the sample library. In subsequent loops, a combination of knowledge‐driven forward and data‐driven reverse inferences was employed to continuously calculate various distributions and coefficients through evidence combination and fusion computing. The entire process can be conceptually represented by the following Equation (1):

(1) $$D_{\mathrm{NEL}}=\sum_{\mathrm{loop}=1}^{\infty }D_{\mathrm{loop}}\left(GH_{P}\right)\;$$

where *GH_P_* indicates a single computed brain map from an independent study, $D_{\mathrm{loop}}\left(GH_{P}\right)$ is the evaluation indicators at one given point of never‐ending learning, including the uncertainty distribution τ and the support coefficient γ. Through evidence combination and fusion computing, these indicators are aggregated to produce $D_{\mathrm{NEL}}$, which provides an overall evaluation of the learning process. Theoretically, this process of never‐ending learning is indefinite if there is sufficient data, along with systematic and explainable experimental planning. In addition, the framework proposed can be mathematically indicated as the following Equation (2):

(2) $$D_{\mathrm{NEL}}^{t}:F_{\mathrm{RULEs}}〈f_{t-1}^{i},\left(x_{t},y_{t}\right),M_{t-1}\rangle \to \langle \left\{f^{i}\right\},M_{t}〉$$

where *F*_RULEs_ indicates our computing framework embedding by rules, $f_{t-1}^{i}$ indicates a method used at *t* − 1, which can be changed or be kept during the subsequent learning processes.

One different point between our framework and other never‐ending learning methods (such as lifelong learning, continuous learning, and continual learning) is the optimized object. In particular, the computing framework *F*_RULEs_ is non‐changed during the learning process, while the computing methods can be changed from $f_{t-1}^{i}$ to {*f^i^*}, and the memory changes from *M*_*t* − 1_ to *M_t_* after an iterative process.

### Systematic and Explainable Experiment Planning

The purpose of systematic experiment planning was to gain a comprehensive understanding of human intelligence and health through a structured approach that mimics human thinking processes. Existing systematic studies often focus on integrating multitask datasets to compare various cognitive functions, treating diverse datasets obtained from different experimental tasks as equally important. However, these approaches overlook the variations in the computational role when modeling multitask datasets, leading to different weights of evidence. To address these issues, the types of experiments based on factors such as cognitive hypotheses and experimental paradigms were differentiated. These experiments were then linked within an experimental template graph, guided by various matching rules based on the function and experiment dimensions of the conceptual Data‐Brain. In an experimental graph, the main experiment (*T*_mae_), which directly corresponds to a target hypothesis, serves as the starting point for systematic experimental planning, while supplementary experiments branching out from the main experiment were continuously designed to support evidence combination and fusion computing. Furthermore, these supplementary experiments (*T*_sue_) can be classified into different types, including similar experiments, parallel experiments, deeper experiments, inspired experiments, missed experiments, subprocessing experiments, and so forth (Figure S2, Supporting Information).

By continuously planning multiple experiments across various experimental types, a hierarchical experimental sequence was generated to guide the integration of related brain resources. The primary challenge lies in planning cognitive experiments within each loop to facilitate never‐ending learning. To address this issue, the study employs the strategy of experimental similarity assessment. Evaluating the similarity among different experiments requires a description of the experimental profile, including factors related to paradigms and stimuli, which could be quantified. These experimental profile‐related factors are represented as the concepts and properties in the experiment dimension of the conceptual Data‐Brain (Figure S1, Supporting Information). In this study, three types of representative factors, the experimental paradigm (EPA), experimental protocol (EPR), and explicit stimulus (SEN), are selected to calculate the experimental similarity as follows:

(3) $$E\left(T_{\mathrm{mae}},T_{\mathrm{sue}}\right)=\frac{1}{\mathrm{the}\;\mathrm{size}\;\mathrm{of}\;\mathrm{facs}}\;\sum_{\mathrm{fac}\in \;\mathrm{facs}}Bin(T_{\mathrm{mae}}^{\mathrm{fac}},T_{\mathrm{sue}}^{\mathrm{fac}})$$

where $E(T_{\mathrm{mae}},T_{\mathrm{sue}})\;$ indicates the similarity of two experiments; facs = {EPA: [categorical design, parametric design, factorial design]; EPR: [event‐related design, block design, mixed design]; SEN: [pictures, digits]; …} reflects the selected factors presented by the dictionary type; $T_{\mathrm{mae}}^{\mathrm{fac}}$ and $T_{\mathrm{sue}}^{\mathrm{fac}}$ indicate one of the factors *fac* from the experiments *T*_mae_ and *T*_sue_, respectively; the function $Bin(T_{\mathrm{mae}}^{\mathrm{fac}},T_{\mathrm{sue}}^{\mathrm{fac}})$ evaluates the consistency between the factors $T_{\mathrm{mae}}^{\mathrm{fac}}$ and $T_{\mathrm{sue}}^{\mathrm{fac}}$, in which *Bin* (·, ·) =  1 if both factors are the same, or *Bin* (·, ·) =  0 if not, which supports intra‐ and inter‐analyses.

### Internal Evidence Learning Based on Intra‐ and Inter‐Analyses

Accompanied by systematic experiment planning, neuroimaging resources corresponding to various experimental details are collected and analyzed to test hypotheses based on two types of analysis strategies:

1. The intra‐analysis strategy constructs an analysis within a specific domain of cognitive functions. When a hypothesis declares a cognitive function domain and a brain pattern (e.g., human reasoning depends on DLPFC.), the intradomain cognitive functions (e.g., child nodes of reasoning, Figure 1B; Figure S1, Supporting Information) are determined according to the function dimension of the conceptual Data‐Brain. This type of analysis increases the level of generality in a given hypothesis.
2. The inter‐analysis strategy works among multiple different domains of cognitive functions. When a hypothesis is made, the interdomain cognitive functions (i.e., the sibling node of the target cognitive function domain in the function dimension of the conceptual Data‐Brain) are determined accordingly. This type of analysis increases the level of specificity in a given hypothesis.

These different analysis types depend on the experimental planning, and then determine different roles of data samples that are computed in each loop. The combination of these two types of analyses reduces relational uncertainty between a cognitive function and multiple brain patterns, and contributes to the stronger hypothesis testing during the forward inference process. Meanwhile, these two types of analyses also have a significant impact on calculating the support coefficient, contributing to the functional interpretations of a brain pattern on different cognitive function domains during the reverse inference process. During this never‐ending learning process, brain‐computed results in each loop are treated as evidence, and then are fused continuously through evidence combination and fusion computing.

### External Evidence Learning Based on Pre‐Trained Language Modeling Techniques

**Figure 7:**
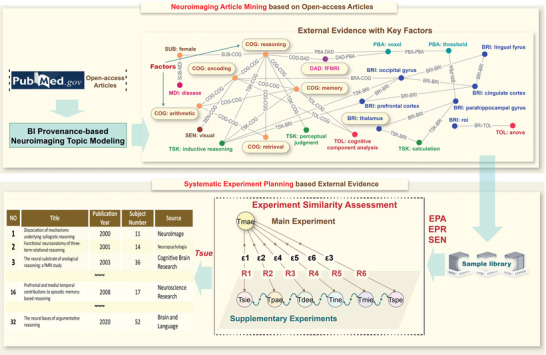
The learning and application of external evidence. The first step is neuroimaging article mining based on open‐access articles, while the second step is systematic experiment planning based on external evidence.

The external evidence comes from outside of our research team. A practical method for large‐scale external evidence learning is through neuroimaging article mining from open‐access articles related to neuroimaging studies (**Figure** **7**). The BI provenance‐based neuroimaging topic modeling method is adopted to extract key factors in experiments and analyses as external evidence. Then, the evidence is stored in the sample library. During learning processes, by the factors‐based experimental similarity assessment, different types of supplementary experiments can be recognized from aligned external evidence and used to support evidence combination and fusion computing.

**Figure 8:**
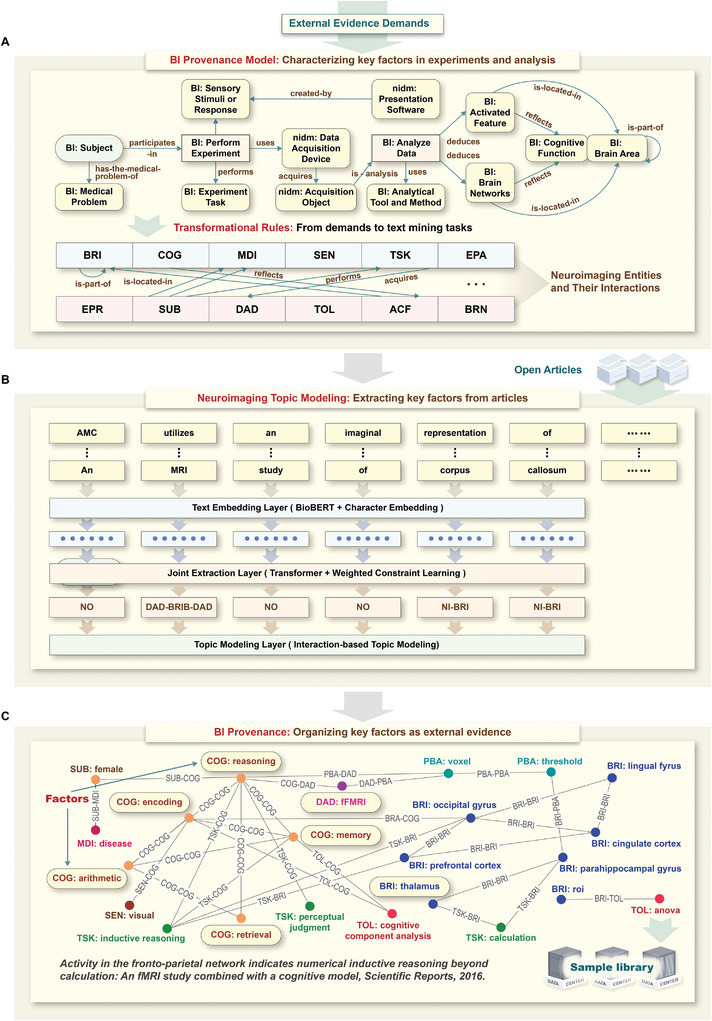
The BI provenance‐based neuroimaging topic modeling method. A) The conceptual BI provenance model is used to capture evidence demands from systematic brain computing. B) The neuroimaging topic modeling pipeline is used to extract external evidence and recognize key factors from open‐access neuroimaging articles. C) BI provenance with key factors is stored in the sample library for evidence combination and fusion computing.

To achieve this, the BI provenance‐based neuroimaging topic modeling method for external evidence learning was used, which involves the following two key steps (**Figure** **8**). The first step involves the task definition of neuroimaging article mining, guided by the BI provenance model that characterizes the key factors of experiments and analyses for systematic experiment planning. To capture the factor demands of systematic experiment planning, a new BI provenance model (Figure 8A) is reconstructed based on the Neuroimaging Data Model (NIDM),^[^ ^47^ ^]^ which consists of three categories with respect to elements: entity (circle), agent (hexagon) and activity (rectangle), and characterizes the key factors in experiments and analyses. This model is then transformed into neuroimaging article mining tasks, which consist of categories of neuroimaging entities and interactions, using the following rules:

1. Entities: Each agent or entity in the BI model is transformed into a category of neuroimaging entities.
2. Interactions: Each relation between agent and entity in the BI model is transformed into a category of neuroimaging interactions. To account for the flexibility of language expression, each potential pair of entities or agents connected to the same activity is considered as a candidate relation. After removing duplicate relations, the remaining candidate relations are also transformed into interaction categories.

Applying these rules, we obtained 12 categories of neuroimaging entities and 55 categories of neuroimaging interactions in the current study (Tables S2 and S3, Supporting Information).

The second step involves interaction‐based neuroimaging topic modeling as shown in Figure 8B, which is the text embedding layer, the joint extraction layer, and the topic modeling layer. The text embedding layer encodes the sentences in neuroimaging articles and constructs text vectors as the input for the subsequent layer. The joint extraction layer adopts a joint deep learning model fusing Transformer and weighted constraint learning to extract neuroimaging entities and their interactions as BI provenance from the input text vectors. The topic modeling layer recognizes research topics as the key factors based on interactions of neuroimaging entities in BI provenance. In particular, the text embedding layer transforms the inputted neuroimaging texts into text vectors by using BioBERT^[^ ^48^ ^]^ and character embedding. The joint extraction layer combines Transformer^[^ ^49^ ^]^ and weighted constraint learning^[^ ^50^ ^]^ to extract neuroimaging entities and interactions as the BI provenance in a few‐shot learning manner. The topic modeling layer identifies key factors of experiments and analyses from extracted neuroimaging entities and interactions, in which the word distribution of the Biterm Topic Model is replaced by the interaction density.

Finally, the BI provenance containing the key factors of experiments and analyses extracted from the neuroimaging text data is stored in the sample library as external evidence (Figure 8C).

### Evidence Combination and Fusion Computing

First, the forward inference‐based evidence combination and fusion computing was designed. The fMRI data were computed to obtain the parametric brain maps, mainly relying on univariate analysis (such as statistical parametric mapping techniques). These computed results will be taken as internal evidence with inferred evidential types and computed weights. More specifically, each piece of evidence was identified as an evidential type for multiview analytical strategies. Moreover, these evidential types with various weight coefficients λ are computed based on the function dimension of the conceptual Data‐Brain, including:

1. Type I‐evidence related to intra‐analyses, where λ  =  1 if the relation of functional domains corresponding to *T*_mae_ and *T*_sue_ in the function dimension of the conceptual Data‐Brain is “descendants”; $\lambda \;=\prod \frac{1}{\mathrm{degree}\;\mathrm{of}\;\mathrm{node}}\;$ if the functional domain of *T*_mae_ is the “ancestor” relationship with that of a *T*_sue_, in which the node is in the shortest path from the functional domain of a *T*_sue_ to the parent of the functional domain of the *T*_mae_ in the function dimension of the conceptual Data‐Brain;
2. Type II‐evidence related to inter‐analyses, where λ  =   − 1 if the relation between *T*_mae_ and *T*_sue_ in the function dimension is different from Type I‐evidence, such as the “sibling” relationship.

This systematic experiment planning combined with the evidential type inference assures evidence combination and fusion computing. At a given loop, the task‐related evidence is fused by calculating the uncertainty distribution τ and interpreting the effect size of brain patterns (e.g., brain region) to a cognitive function underlying a specific goal hypothesis, which is given by:

(4) τloop(GHP)=∑i=1R(Ti)⊆GHPNloopλ×R(Ti),(−∞<τ<∞)

where τ_loop_(*GH_P_*) indicates an uncertainty brain map at a loop; *N*_loop_ indicates the amount of loops corresponding to various evidence from the main and supplementary experiments; and *R*(*T_i_*) indicates the computing results of evidence related to the experiment *T_i_* from an independent study.

Second, the reverse inference‐based evidence combination and fusion computing are performed by integrating the multivariate pattern analysis methods. During this learning process, different machine learning and deep learning methods defined in the analysis dimension of the conceptual Data‐Brain can be selected to evaluate the distinctions of brain patterns to different experimental conditions. Herein, the support vector machine method with grid search was performed to discriminate different cognitive states for hypothesized brain regions in each loop. The recognized results would taken as support coefficients, which were computed by evidence combination and fusion computing from three views of cognitive states, depending on the complexity, condition, and component. For the view of complexity, the predictive results are used to test the information‐processing capability of a brain pattern, corresponding to experimental tasks with varied complexity (such as the complex task vs. the simple task). For the view of the condition, a brain pattern is tested by discriminating relations between the same‐level components of interest (such as addition vs. subtraction within a mental arithmetic task). For the view of the component, a brain pattern was tested by discriminating relations between a component of interest and the baseline component (such as number induction vs. number judgment within human reasoning). Considering the experimental characteristics, a greater difference in experimental tasks may induce a greater difference in brain activity patterns, and then impact the classification effects. Hence, the weights are bounded by different predictive modes Φ, as follows:

(5) $$\left\{\begin{array}{cc} \alpha (X), & \mathrm{if}\;X=\mathrm{the}\;\mathrm{complexity}\;\mathrm{level}\;\mathrm{based}\;\mathrm{classification}.\\ \alpha (Y), & \mathrm{if}\;Y=\mathrm{the}\;\mathrm{condition}\;\mathrm{level}\;\mathrm{based}\;\mathrm{classification}.\\ \alpha (Z), & \mathrm{if}\;Z=\mathrm{the}\;\mathrm{component}\;\mathrm{level}\;\mathrm{based}\;\mathrm{classification}. \end{array}\right.\;$$

where α(·) indicates the computed weights, α(*X*)  ≈  α(*Y*)  >  α(*Z*), and α(*X*) +  α(*Y*) +  α (*Z*) =  1. Hence, the predictive results of multiple evidence corresponding to the intra‐analysis can be fused to answer the question: how to interpret the information‐processing capability of a specific brain pattern (BRP) to different cognitive functions (CFD)? Its effect indicator $D_{\mathrm{NEL}}$ is measured by the support coefficient γ, which is calculated by:

(6) $$\begin{array}{c} \gamma \left(BRP\to CFD\right)=\sum_{\Phi \in \{X,Y,Z\}}\left(\frac{\sum_{i=1}^{N(\Phi )}\alpha \left(\Phi \right)\times P\left(\Phi \right)}{N(\Phi )}\right),\left(0\leq \gamma \leq 1\right) \end{array}$$

where N(Φ) is the number of the predictive mode Φ, and P(Φ) indicates the predictive results (such as the classification accuracy obtained by machine learning and deep learning methods) corresponding to the predictive mode Φ.

### Human‐In‐The‐Loop Interactive Learning

The human‐in‐the‐loop^[^ ^51^, ^52^ ^]^ mechanism was an integral part of the never‐ending learning paradigm designed to enhance its robustness and scalability. Throughout the learning processes, investigators can interactively access each loop, including setting requests, refining resources, and screening resources:

1. Request setting was an individualized process within the conceptual Data‐Brain. In this stage, investigators could constrain the graph of cognitive components in the function dimension based on their research interests and background knowledge.
2. During the experimental stage, investigators can design task‐related parameters of interest, such as experimental paradigms, experimental protocols, and explicit stimuli.
3. In the data dimension, investigators can design data‐related parameters of interest, such as the data modality in fMRI, the data state in raw and text data, and the subject type in healthy individuals.
4. In the analysis dimension, computing details can be designed individually. For example, if the general linear model is used in the forward inference, the related parameters will include the statistical *P* value, corrected methods, and the size of the cluster selected from the set {10, 20, 30, 50, 100, 150, …}. Moreover, when machine learning methods are used in the reverse inference, the selection of classification models is necessary.
5. Resource refining occurs at updating stages (such as add, modify, delete, and query operations) of the sample library and the conceptual Data‐Brain. For example, during those processes of mapping the external evidence to the internal sample library, an investigator can ensure the correctness of the information and add any missing information.

Furthermore, an investigator is indispensable in extending and refining the personal computing graph from the global graph surrounding the conceptual Data‐Brain. For this purpose, the pre‐trained language models are considered to construct a global graph from raw text to knowledge graph easily.^[^ ^53^, ^54^ ^]^ In this work, an end‐to‐end pre‐trained language model is used to extract entities and relations from raw text data to a global graph, that is the “Relation Extraction By End‐to‐end Language (REBEL)” generation,^[^ ^55^ ^]^ especially in the topic of brain research. Finally, the dynamic personal computing graph is designed individually to guide the multi‐source data extraction, evidence combination, and systemic fusion computing over time through prompts.

## Conflict of Interest

The authors declare no conflict of interest.

## Author Contributions

H.K. and J.C. contributed equally to this work. H.K., N.Z., and P.L. conceived the concept and devised the study. H.K., J.C., P.L., and N.Z. developed the method. P.L., N.Z., J.C., H.K., and L.C. provided data. H.K. and J.C. performed the analysis. X.T., K.I., and H.M. helped with the experiments and provided helpful discussions. H.K. wrote the original manuscript. All authors reviewed and edited the manuscript.

## Supporting information

Supporting Information

## Data Availability

The data that support the findings of this study are available in the supplementary material of this article.
