# Supplementary material for: Never‐Ending Learning for Explainable Brain Computing
Source: Adv Sci (Weinh). 2024 Apr 11;11(24):2307647. doi: 10.1002/advs.202307647 (PMC11200082; doi:10.1002/advs.202307647)
Supplement: Supplementary file 1 — Supporting Information [file ADVS-11-2307647-s001.pdf]

ADVANCED SCIENCE
Open Access

Supporting Information

for *Adv. Sci.*, DOI 10.1002/advs.202307647

Never-Ending Learning for Explainable Brain Computing

Hongzhi Kuai, Jianhui Chen, Xiaohui Tao, Lingyun Cai, Kazuyuki Imamura, Hiroki Matsumoto, Peipeng Liang and Ning Zhong**

Supplementary Materials for

Never-Ending Learning for Explainable Brain Computing

Hongzhi Kuai and Ning Zhong* *et al.*

*Corresponding author. Email: zhong@maebashi-it.ac.jp

This PDF file includes:

Supplementary Text
Figure S1 to S2
Table S1 to S6
References 1 to 13

Supplementary Text

The workflows of NEL (never-ending learning)-Explainable Brain Computing Framework are given as follows.

NEL-based Explainable Brain Computing Framework

Resource Layer:
- *Knowledge (K)*: global (learned by automatic methods) and personal (defined by hand-crafted methods) graphs, etc.
- *Information (I)*: provenances (corresponding to data, analyses, and study findings, etc.)
- *Data (D)*: raw data (structural MRI, task-state fMRI); parametric mappings (statistical and machine learning methods); and coordinates (single and multiple studies), etc.

Learning Layer:
- *Internal Evidence Learning Component*:
  - Analyze brain images from raw data to parametric maps
  - Transform study results from coordinates to parametric maps
- *External Evidence Learning Component*:
  - Construct the global graph using large-language models and domain corpus
  - Identify the data and analysis provenances from the domain corpus automatically
- *Evidence Combination and Fusion Computing*:
  - While ending condition is not achieved do:
    - Sample brain data based on rules and personal graphs
    - Data computing and alignment to a predefined brain template
    - Fuse selected brain data based on knowledge and meta information
  - End while

Interaction Layer:
- Initialize the hypothetical details, the aspects that the users want to observe and verify, such as experimental preference and personal graphs
- Determine a start-up experiment from a recommended list

The sequence of uncertainty brain maps with experimental details

Resource Integration Based on Knowledge (K)-Information (I)-Data (D) Architecture. In this study, we constructed a sample library to facilitate the integration and operation of brain big data across multiple resource layers, following the KID architecture [1,2]. Specifically, the knowledge layer consists of the conceptual Data-Brain, which encompasses conceptual elements from four dimensions: function, experiment, data, and analysis, representing the systematic brain investigation processes. These elements and their relationships support evidential inference, learning, and computing. The information layer corresponding to the data and analysis provenances serves as a bridge between knowledge and data layers, facilitating evidential traceability. On one hand, the factors in the provenance of the information layer inherit the scopes of the knowledge layer, driving neuroimaging topic modeling from external evidence learning. On the other hand, the provenance of the information layer enables the tracking of various brain data in the data layer, facilitating systematic brain decoding-oriented data integration and sampling. Consequently, brain big data can be interconnected within a unified graph structure with high interactivity. The data layer covers the multitype resources, such as raw data, processed data, and results of studies to meet requirements from both inference perspectives of internal and external evidence.

MRI Preprocessing. The framework integrates several preprocessing tools (including the SPM12 toolbox, https://www.fil.ion.ucl.ac.uk/spm/ and the fMRIPrep toolbox [3], https://fmriprep.org/en/stable/) currently available for fMRI investigation: (1) slice-timing correction; (2) estimation of rigid-body motion; (3) co-registration of the anatomical image in the Montreal Neurological Institute (MNI) space; (4) individual co-registration between anatomical and functional images; (5) resampling; (6) spatial smoothing with an isotropic Gaussian kernel; and (7) temporal filtering.

fMRI-related Computing and Analysis Methods. In each loop of the forward inference on the ROI analysis, general linear model (GLM) and multivariate pattern analyses were conducted to determine task-related activation/deactivation clusters from the hypothesized brain regions [4,5]. Task-related ROIs were determined on the contrast of interest, as shown in Table S1 of Supplementary Information: numerical placement puzzles vs. symbolic placement puzzles in D1, complex numerical inductive reasoning vs. simple numerical inductive reasoning in D4, rule induction vs. perceptual judgment in D51, rule application vs. perceptual judgment in D52, arithmetic addition vs. arithmetic subtraction in D6, numerical inductive reasoning vs. perceptual judgment in D71, calculation vs. perceptual judgment in D72, numerical inductive reasoning vs. perceptual judgment in D81, and letter inductive reasoning vs. perceptual judgment in D82. During the reverse inference, support vector machines with grid search (including the parameters gamma = {0.001, 0.01, 0.1} and C = {1,10,100} of the Radial Basis Function kernel) were selected to discriminate between different cognitive states in each loop.

Text Data Collection. We collected neuroimaging articles from PubMed, which is the premier research database for the health science. Because PubMed has a large scale and does not fully support online full-text crawling, we choose more detailed keywords based on the research objectives of this article. Only neuroimaging articles related to human reasoning studies were gathered using search strings with the keywords "(reasoning) AND (fMRI) OR (reasoning) AND (functional MRI) OR (reasoning) AND (functional magnetic resonance imaging) OR (inductive reasoning) AND (fMRI) OR (inductive reasoning) AND (functional MRI) OR (inductive reasoning) AND (functional magnetic resonance imaging) OR (deductive reasoning) AND (fMRI) OR (deductive reasoning) AND (functional MRI) OR (deductive reasoning) AND (functional magnetic resonance imaging)" in the full texts. After excluding invalid article, such as the meta-analysis articles and the articles without reported coordinates, 44 full-text articles were downloaded. We also performed the BI provenance-based neuroimaging topic modeling

method on these articles and then stored the extracted BI provenance with key factors in the sample library.

Resource Constraint Analysis. Currently, we verified the framework in the scenario of analyzing multi-task brain images to understand high-order cognition, while it still works if the resources are organized and the purpose is defined to satisfy the following principles. In particular, the resources are organized in the knowledge-information-data architecture, covering multisource text and neuroimage. However, considering the data constraints, the framework might struggle with some specific scenarios:

- Firstly, the framework on handling multi-scale data, such as genetics, will face challenges. The core challenge is alignment. Accordingly, the framework should enhance its flexibility and alignment ability on integrating task-free data, multiscale data, and so forth.
- Secondly, the interactions and associations among cognitive functions during conditions and diseases are other valuable topics, in which the structural and functional alignment problem needs to be further investigated to reduce the gap between cognitive and clinical findings.
- Thirdly, we aim to further confront challenges in translational research to make it available in clinical practices. Therefore, we need to further integrate other technologies, such as brain stimulation, providing a novel perspective for decoding cognitive mechanisms.

We encourage more scientists from different backgrounds to explore and recognize its value in various scenarios, contributing to a more profound understanding.

Computational Complexity Analysis. In the NEL-based explainable brain computing framework, these complexities of computational components need to be concerned about as follows:

1. The computational complexity of the conceptual Data-Brain construction via the language models, such as the learning processes from text data to global graphs;
2. The computational complexity of the internal evidence learning, such as the analysis processes of parametric maps;
3. The computational complexity of the internal evidence learning, such as the mapping processes from the reported coordinates to parametric maps;
4. The computational complexity of the external evidence learning, such as the mining processes of the named entity recognition;
5. The evidence combination and fusion computing of the internal and external evidence, including the brain data selection, data alignment to brain template, and the data fusion processes.

The computing components from 1 to 4 have the dynamic complexity, depending on the used methods in the framework. In addition, the upper bounds of the computational complexity on the fifth component “Evidence Combination and Fusion Computing” can be given as follows. Herein, N is the number of computational samples, and P is the number features with respect to the brain template scale. The computational complexity is mainly from the data alignment operation to a predefined brain template, and the fusing operation for selected brain data. As we do not need pretrain the data in the framework, the approximation of the computational complexity is be given with $O(NP)$.

Computational Sensitivity Analysis. On basis of the KID architecture, the KID loop supports never-ending learning, which tests the goal hypothesis along with the generation, evolution and learning of multiple sources surrounding knowledge, information and data, continuously. At the early iterations, the proposed brain computing framework is relatively sensitive to the quality of the input data. High quality data will lead to reliable results quickly. However, as the number of iterations increases, the incorporation of more and more internal and external

evidence will reduce sensitivity to data quality. To address this issue, we design the operating rules to reduce the impact from low-quality data. In details, the framework performs internal evidence with high confidence (from intra-experiment to inter-experiment evidence) firstly, and then performs external evidence with relatively low confidence (from intra-experiment to inter-experiment evidence).

Technicial Summarization. In the details of the resource layer, the data are organized by the Brain Imaging Data Structure (BIDS) standard; the information is organized in relational tables; and the knowledge is organized by the Resource Description Framework (RDF) in triple tables. During the internal evidence process, the raw brain images are analyzed by the general liner model and multivariate pattern analysis methods, while the extracted peak coordinates are mapped to the standard brain template via the Python nilearn library. During the external evidence learning process, a global graph is constructed by the "Relation Extraction By End-to-end Language (REBEL)" framework[6], as shown in Figure S1 of Supplementary Information, guiding the construction of personal subgraphs to execute systematic computing operations, together with the human-in-the-loop operations. In addition, the provenances are learned by the Neuroimaging Data Model (NIDM) and BioBERT, identifying the topics of studies through interaction-based neuroimaging topic modeling. These topic-tagged studies with reported results will be further computed during the never-ending learning processes. To perform evidence combination and fusion computing, the program is simulated by the Python libraries of nibabel, nilearn, and so forth. In the interaction layer, the human can determine the experimental preference and the start-up evidence corresponding to a proposed hypothesis.

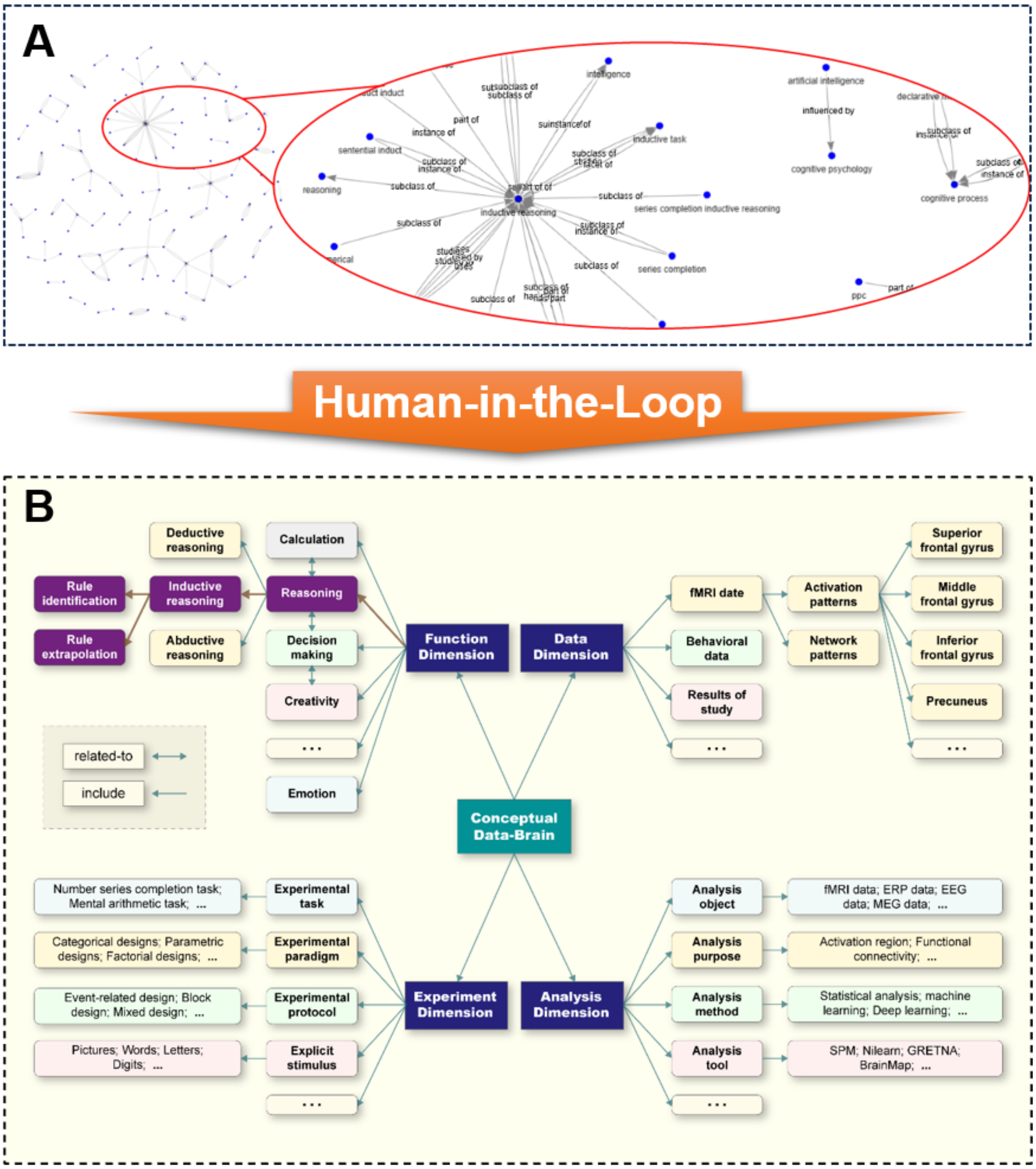

Figure S1.

Construction of the conceptual Data-Brain. A. Global graph is constructed by pre-trained language modeling techniques, from raw text data to structural knowledge graph. B. A personal graph is constructed by reorganizing the global graph with prompts, including the four dimensions of function, experiment, data and analysis, corresponding to the four aspects of the Brain Informatics methodology: systematic investigations of complex brain science problems; systematic design of cognitive experiments; systematic brain data management; and systematic brain data analysis and simulation.

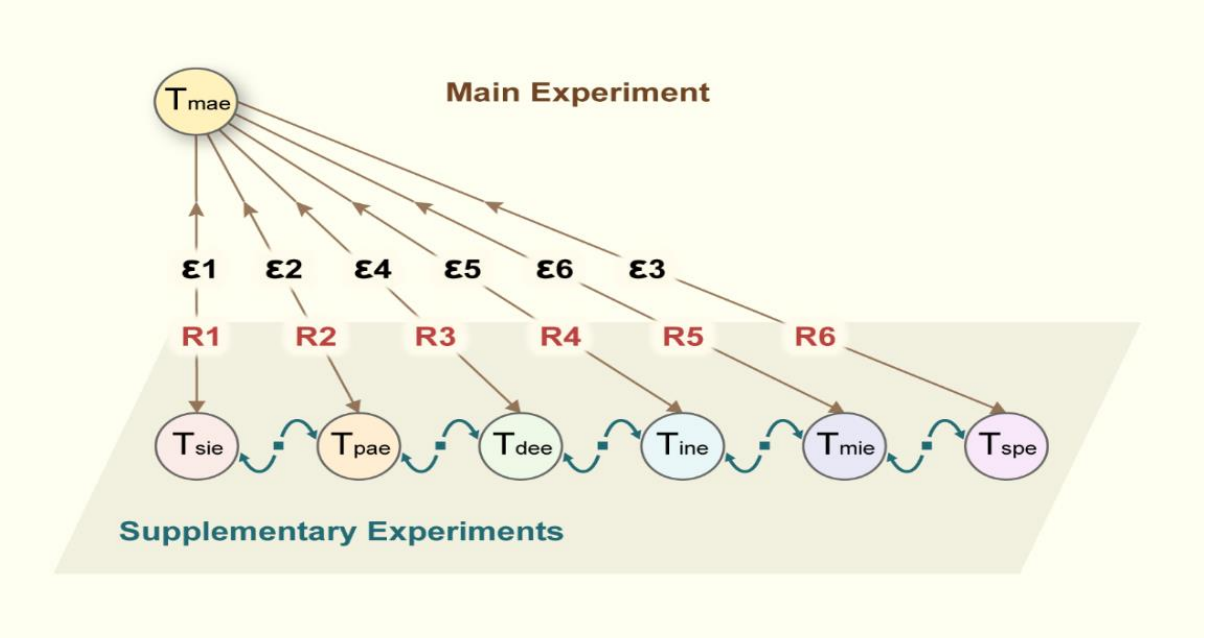

Figure S2.

The template graph of systematic experimental planning. The main experiment (T_{mae}) that corresponds directly to the goal hypothesis is a starting point for systematic experimental planning, and the supplementary experiments are driven by the main experiment as continuous support of evidence combination and fusion computing. The supplementary experiments are further defined as various experimental types, including the similar experiment (T_{sie}), parallel experiment (T_{pae}), deeper experiment (T_{dee}), inspired experiment (T_{ine}), missed experiment (T_{mie}) and subprocessing experiment (T_{spe}). The reasoning rules of various experimental types are described as follows. R1: If an experiment is identified as a similar experiment, its task shares similar factors with T_{mae} in the function and experiment dimensions. Under these circumstances, T_{sie} and T_{mae} may have different factors in practice, such as device and brain image parameters from multiple data centers. R2: If an experiment is identified as a parallel experiment, its task shares similar factors in the function dimension with T_{mae} but may have different factors in the experiment dimension, such as digits and symbols. R3: If an experiment is identified as a deeper experiment, its task is used to further explore hidden mental processes related to T_{mae}, but corresponding to different hypotheses with other factors in the function dimension. For instance, calculation-related cognitive activity can be studied through arithmetic tasks. However, such a task is not only relevant to calculation processing but also to the integration of numerical and symbolic processing that must be further considered. R4: If an experiment is identified as the inspired experiment, its task is used to test the goal hypothesis involving different factors in the function dimension from T_{mae} but shares similar factors in the experiment dimension with T_{mae}. R5: If an experiment is identified as a missed experiment, its task does not satisfy the aforementioned criteria but evokes similar brain activities (such as patterns and indicators) with T_{mae}. R6: If an experiment is identified as the subprocessing experiment, its task is used to test the goal hypothesis-related single aspect within a dual-task paradigm. For instance, an experimental design for the association study of emotion and calculation may be regarded as two separate tasks to test emotional and calculation hypotheses, respectively. The experimental similar degree $\{\varepsilon_i | 1 \le i \le 6\}$ between the main experiment and its supplementary experiment is computed by experimental similarity assessment (see the systematic experimental planning approach in Section 4.2).

Table S1.
A fragment of the sample library with the symbiosis of internal evidence. In the sample library, each piece of evidence is regarded as a chain of evidence that contains the functional neuroimaging data, the results of studies, and their context, such as the study purpose, experimental design and processing methods.

ID	COG	EPA	EPR	SEN	Subjects (#)
D1 [7]	Reasoning	Factorial	Event-related	Digits, Symbols	Healthy (11)
D2 [7]	Social	Factorial	Event-related	Faces, Shapes	Healthy (30)
D3 [8]	Emotion, Calculation	Factorial	Block	Pictures	Healthy (13), MDD (13)
D4 [9]	Reasoning	Factorial	Event-related	Digits	Healthy (15)
D51 [10]	Reasoning-Component 1	Categorical	Event-related	Digits	Healthy (20)
D52 [10]	Reasoning-Component 2	Categorical	Event-related	Digits	Healthy (20)
D6 [11]	Calculation	Categorical	Block	Digits, Symbols	Healthy (22)
D71 [12]	Reasoning	Categorical	Block	Digits, Symbols	Healthy (15)
D72 [12]	Calculation	Categorical	Block	Digits, Symbols	Healthy (15)
D81 [13]	Reasoning	Factorial	Block	Digits	Healthy (23)
D82 [13]	Reasoning	Factorial	Block	Letters	Healthy (23)
		…			

ID: Identifier of the Experimental Data in the Sample Library; COG: cognitive function; EPA: experimental paradigm; EPR: experimental protocol; SEN: explicit stimulus; #: number of subjects; MDD: major depressive disorder.

Table S2.

Twelve categories of neuroimaging entities obtained from the BI provenance model. These entities indicate the key factors in experiments and analyses that can be used for evidence combination and fusion computing.

Category	Definition	Example
Brain Area (BRI)	Brain area is an area in the human cortex that responds to one or several cognitive tasks during the neuroimaging study.	Motor, language, and learning: functional magnetic resonance imaging of the cerebellum.
Cognitive Function (COG)	Cognitive function is an ability of the brain to process information during the neuroimaging study.	Control of goal-directed and stimulus-driven attention in the brain.
Medical Problem (MDI)	The medical problem is an abnormal symptom of subjects during the neuroimaging study.	Clinical and experimental study on adrenomedullin in acute myocardial infarction.
Explicit Stimulus (SEN)	The explicit stimulus is a kind of sensory channel of subjects presented by stimuli during the neuroimaging study.	The simulation and analysis of the biological olfactory neural model.
Experimental Task (TSK)	The experimental task is a cognitive task that the subject needs to complete during the neuroimaging study.	The external datasets for the color-word stroop task.
Experimental Paradigm (EPA)	The experimental paradigm is an experimental setup (i.e., a way to conduct a certain type of experiment) that is defined by certain fine-tuned standards and often has a theoretical background, including categorical designs, parametric designs, and factorial designs.	Due to the involvement of two factors in the present study, the group-level analysis was implemented based on a 2 by 3 factorial design.
Experimental Protocol (EPR)	The experimental protocol involves the management of variables, their presentation, the assignment of respondents, and the statistical procedures of analysis, especially for event-related design, block design and mixed design.	Within each session, stimuli were presented randomly in an event related design.
Subject (SUB)	The subject is a person who completes the cognitive task during the neuroimaging study.	The patient indicates whether or not the word was shown previously.
Data Acquisition Device (DAD)	The data acquisition device is a kind of professional equipment that is used to record the psychological or physiological data of subjects during the neuroimaging study.	Application of positron emission tomography in the central nervous system.
Analytical Tool and Method (TOL)	The analytical tool and method are a data analytical algorithm or software, which is used to mine experimental data during the neuroimaging study.	Data processing and analysis of MRI based on principal component analysis.
Activated Feature (ACF)	The activated feature is a brain response that is mined from experimental data during the neuroimaging study.	The peak of the activation coordinate began to decrease.
Brain Networks (BRN)	The brain networks are a kind of brain responses that are mined from experimental data during the neuroimaging study.	An fMRI study of deactivation and default mode network activity in human brain.

Table S3.
Partial categories of neuroimaging interactions obtained from the BI provenance model, where interaction indicates semantic relations between entities.

Category	Type ID	Definition
is-part-of	BRI-BRI	The "is-part-of" is the interaction between two "Brain Area" entities, which indicates the inclusion relation between brain areas.
reflect	COG-ACF	The "reflect" is the interaction between the "Cognitive Function" entity and the "Activation Feature" entity, which indicates "Activation Coordinate" reflects the "Cognitive Function" in the cognitive research.
is-located-in	ACF-BRI	The "is-located-in" is the interaction between the "Activation Feature" entity and the "Brain Area" entity, which indicates the brain response appears in the "Brain Area".
perform	SUB-TSK	The "perform" is the interaction between the "Subject" entity and the "Experimental Task" entity, which indicates the "Subject" performs the "Experimental Task" in the neuroimaging study.
has-the-medical-problem-of	SUB-MDI	The "has-the-medical-problem-of" is the interaction between the "Subject" entity and the "Medical Problem" entity, which indicates the "Subject" is suffering from the "Medical Problem".
acquire	TSK-DAD	The "acquire" is the interaction between the "Experimental Task" entity and the "Data Acquisition Device" entity, which indicates researchers collect brain data related to the "Experimental Task" through the "Data Acquisition Device".
		…

Table S4.
Human reasoning-related neuroimaging articles from PubMed and the PLOS series, which are recognized from the sample library based on similarity assessment during the systematic experimental planning process.

NO	Title	Publication Year	Subject Number	Source
1	Dissociation of mechanisms underlying syllogistic reasoning	2000	11	NeuroImage
2	Functional neuroanatomy of three-term relational reasoning	2001	14	Neuropsychologia
3	The neural substrate of analogical reasoning: a fMRI study	2003	36	Cognitive Brain Research
4	Reasoning and working memory: common and distinct neuronal processes	2003	12	Neuropsychologia
5	Differential involvement of left prefrontal cortex in inductive and deductive reasoning	2004	15	Cognition
6	The cerebellum and decision making under uncertainty.	2004	8	Cognitive Brain Research
7	The effect of social content on deductive reasoning: An fMRI study	2005	55	Human Brain Mapping
8	fMRI evidence for a three-stage model of deductive reasoning	2006	12	Cognitive Neuroscience
9	Neural correlates of superior intelligence: Stronger recruitment of posterior parietal cortex	2006	36	NeuroImage
10	Frontopolar cortex mediates abstract integration in analogy	2006	27	Brain Research
11	An fMRI investigation of the role of the basal ganglia in reasoning	2007	22	Brain Research
12	Neural basis of generation of conclusions in elementary deduction.	2007	14	NeuroImage
13	Distinct neural substrates for deductive and mathematical processing	2008	16	Brain Research
14	Developmental shifts in fMRI activations during visuospatial relational reasoning	2009	16	Brain and Cognition
15	Differential patterns of cortical activation as a function of fluid reasoning complexity	2009	20	Human Brain Mapping
16	Prefrontal and medial temporal contributions to episodic memory-based reasoning	2008	17	Neuroscience Research
17	The dynamics of deductive reasoning: An fMRI investigation	2009	12	Neuropsychologia
18	Emotional and cognitive stimuli differentially engage the default network during inductive reasoning	2011	20	Social Cognitive and Affective Neuroscience
19	Long-range functional interactions of anterior insula and medial frontal cortex are differently modulated by visuospatial and inductive reasoning tasks	2013	22	NeuroImage

20	Contradictory Reasoning Network: An EEG and fMRI study	2014	13	PLOS series
21	Common and dissociable neural correlates associated with component processes of inductive reasoning	2011	20	NeuroImage
22	Relational complexity modulates activity in the prefrontal cortex during numerical inductive reasoning: An fMRI study	2014	20	Biological Psychology
23	Neural efficiency as a function of task demands	2014	58	Intelligence
24	Modafinil alters intrinsic functional connectivity of the right posterior insula: a pharmacological resting state fMRI study	2014	26	PLOS series
25	Tracking functional brain changes in patients with depression under psychodynamic psychotherapy using individualized stimuli	2014	35	PLOS series
26	Decreased peripheral and central responses to acupuncture stimulation following modification of body ownership	2014	17	PLOS series
27	Task and resting-state fMRI task and resting-state fMRI reveal altered salience responses to positive stimuli in patients with major depressive disorder	2016	38	PLOS series
28	Activity in the fronto-parietal network indicates numerical inductive reasoning beyond calculation: An fMRI study combined with a cognitive model	2016	15	Scientific Reports
29	Neuroscientific insights into the development of analogical reasoning	2018	138	Developmental Science
30	Decoding rule search domain in the left inferior frontal gyrus	2018	13	PLOS series
31	The neural bases of argumentative reasoning	2020	52	Brain and Language

Table S5.

Three types of representative factors corresponding to the 31 human reasoning-related neuroimaging articles for the external evidence learning during the never-ending learning process.

NO	GOG	EPA	EPR	SEN	Subjects (#)
1	Reasoning	Factorial	Event-related	Sentences	Healthy (11)
2	Reasoning	Factorial	Event-related	Sentences	Healthy (14)
3	Reasoning	Factorial	Block	Words	Healthy (10),
4	Reasoning, Memory	Factorial	Block	Sentences	Healthy (22)
5	Reasoning	Factorial	Event-related	Sentences	Healthy (16)
6	Reasoning	Factorial	Block	Balls, Symbols	Healthy (8)
7	Reasoning	Categorical	Block	Imaging	Healthy (55)
8	Reasoning	Factorial	Block	Letters	Healthy (12)
9	Reasoning	None	Event-related	Pictures	Healthy (33)
10	Reasoning	None	Event-related	Words	Healthy (19)
11	Reasoning	None	Event-related	Pictures	Healthy (22)
12	Reasoning	Factorial	Event-related	Sentences	Healthy (14)
13	Reasoning	Factorial	Event-related	Sentences, Math Equals	Healthy (16)
14	Reasoning	Factorial	Block	Vocabulary	Healthy (16)
15	Reasoning	Factorial	Event-related	Symbols	Healthy (20)
16	Episodic Memory, Reasoning	Categorical	Event-related	Scenes	Healthy (17)
17	Reasoning	Categorical	Block	Word	Healthy (22)
18	Reasoning	Factorial	Event-related	Sentences	Healthy (20)
19	Reasoning	Factorial	Event-related	Sentences, Figure	Healthy (22)
20	Reasoning	None	Event-related	Sentences	Healthy (13)
21	Reasoning	Factorial	Event-related	Digits	Healthy (15)
22	Reasoning	Categorical	Event-related	Digits	Healthy (20)
23	Reasoning	Categorical	Event-related	Number	Healthy (20)
24	Reasoning	Factorial	Event-related	Number	Healthy (58)
25	Reasoning	~	~	~	Healthy (36)
26	Reasoning,	Categorical	Block	Letters	Healthy (21)
27	Reasoning	Factorial	Event-related	Number	Healthy (13)
28	Emotion, Calculation	Factorial	Block	Pictures	Healthy (13), MDD (13)
29	Reasoning, Calculation	Categorical	Block	Digits, Symbols	Healthy (15)
30	Reasoning	Factorial	Event-related	Picture	Healthy (138)
31	Reasoning	Categorical	Block	Letters	Healthy (13)

Table S6.
The learned τ-Values in the peak coordinates selected from the last loop LOOP-23 are given throughout all learned loops, where the selection conditions of peaks are Voxels > 500 and τ-Values > 0.

Peak Selected by LOOP-23 (Voxels > 500; τ-Values > 0)	LOOP-1 (τ-Values)	LOOP-2 (τ-Values)	LOOP-3 (τ-Values)	LOOP-4 (τ-Values)	…
**(44, 30, 18)**	0.66427	0.66427	0.66427	1.16770	
**(-44, 24, 34)**	0.62150	0.62150	0.62150	1.13582	
**(-40, -32, 50)**	0.00000	0.00000	0.57217	0.86931	
**(-42, 44, 10)**	0.44631	0.44631	0.44631	0.96351	…
**(36, -24, 46)**	0.62046	0.62046	0.62046	0.96219	
**(26, 52, 6)**	0.48743	0.48743	0.48743	0.83849	
**(-46, 52, -8)**	0.00000	0.00000	0.00000	0.40777	

LOOP-5 (τ-Values)	LOOP-6 (τ-Values)	LOOP-7 (τ-Values)	LOOP-8 (τ-Values)	LOOP-9 (τ-Values)	LOOP-10 (τ-Values)	…
1.16770	1.16770	1.16770	1.586090	1.58609	1.58609	
1.53983	1.53983	1.53983	1.53983	1.53983	1.56694	
0.86931	0.86931	0.86931	0.86931	0.86931	1.26787	
0.96351	0.96351	0.96351	1.01068	1.01068	1.01068	…
0.96219	0.96219	0.96219	0.96219	0.96219	0.96219	
0.83849	0.83849	0.83849	0.83849	0.83849	0.83849	
0.40777	0.40777	0.40777	0.40777	0.76373	0.76373	

LOOP-11 (τ-Values)	LOOP-12 (τ-Values)	LOOP-13 (τ-Values)	LOOP-14 (τ-Values)	LOOP-15 (τ-Values)	LOOP-16 (τ-Values)	…
1.58609	1.58609	1.58609	1.54124	1.54124	1.54124	
1.56694	1.56694	1.56694	1.56694	1.56694	1.56694	
1.26787	1.26787	1.26787	1.26787	1.26787	1.26787	
1.01068	1.01068	1.01068	1.01068	1.01068	1.01068	…
0.96219	0.96219	0.96219	0.96219	0.96219	0.96219	
0.83849	0.83849	0.83849	0.83849	0.83849	0.83849	
0.76373	0.76373	0.76373	0.76373	0.76373	0.76373	

LOOP-17 (τ-Values)	LOOP-18 (τ-Values)	LOOP-19 (τ-Values)	LOOP-20 (τ-Values)	LOOP-21 (τ-Values)	LOOP-22 (τ-Values)	LOOP-23 (τ-Values)
1.48275	1.48275	1.48275	1.48275	1.48275	1.48275	1.48275
1.56694	1.56694	1.56694	1.48436	1.37115	1.37115	1.37115
1.26787	1.26787	1.26787	1.26787	1.26787	1.26787	1.26787
1.01068	1.01068	1.01068	1.01068	1.01068	1.01068	1.01068
0.96219	0.96219	0.96219	0.96219	0.96219	0.96219	0.96219
0.83849	0.83849	0.83849	0.83849	0.83849	0.83849	0.83849
0.76373	0.76373	0.76373	0.73006	0.73006	0.73006	0.73006

References

1. Kuai, H. *et al.* Multi-source brain computing with systematic fusion for smart health. *Information Fusion* **75**: 150-167 (2021).
2. Kuai, H. & Zhong, N. The extensible Data-Brain model: Architecture, applications and directions. *Journal of Computational Science* **46**: 101103 (2020).
3. Esteban, O., Markiewicz, C.J., Blair, R.W. *et al.* fMRIPrep: a robust preprocessing pipeline for functional MRI. *Nature Methods* **16**, 111–116 (2019).
4. Friston, K. J. *et al.* Statistical parametric maps in functional imaging: a general linear approach. *Human Brain Mapping* **2**: 189-210 (1994).
5. Kriegeskorte, N. *et al*. Information-based functional brain mapping. *Proceedings of the National Academy of Sciences* **103**: 3863-3868 (2006).
6. Cabot, P. L. H., & Navigli, R. (2021, November). REBEL: Relation extraction by end-to-end language generation. In Findings of the Association for Computational Linguistics: EMNLP 2021 (pp. 2370-2381).
7. Kuai, H. et al. THINKING-LOOP: The semantic vector driven closed-loop model for brain computing. *IEEE Access* **8**, 4273-4288 (2020).
8. Yang, Y. *et al.* Task and resting-state fMRI reveal altered salience responses to positive stimuli in patients with major depressive disorder. *PLoS One* **11**, e0155092 (2016).
9. Zhong, N. *et al.* Neural substrates of data-driven scientific discovery: An fMRI study during performance of number series completion task. *Science China Life Sciences* **54**, 466-473 (2011).
10. Jia, X. *et al.* Common and dissociable neural correlates associated with component processes of inductive reasoning. *NeuroImage* **56**, 2292-2299 (2011).
11. Yang, Y. *et al.* The functional architectures of addition and subtraction: Network discovery using fMRI and DCM. *Human Brain Mapping* **38**, 3210-3225 (2017).
12. Liang, P., Jia, X., Taatgen, N. A., Borst, J. P. & Li, K. Activity in the fronto-parietal network indicates numerical inductive reasoning beyond calculation: An fMRI study combined with a cognitive model. *Scientific Reports* **6**, 1-10 (2016).
13. Liang, P., Jia, X., Taatgen, N. A., Zhong, N. & Li, K. Different strategies in solving series completion inductive reasoning problems: An fMRI and computational study. *International Journal of Psychophysiology* **93**, 253-260 (2014).
